# Phylogenetic Analysis of Wild Species and the Maternal Origin of Cultivars in the Genus *Lilium* Using 114 Plastid Genomes

**DOI:** 10.3389/fpls.2022.865606

**Published:** 2022-07-22

**Authors:** Qing Duan, Fang Liu, Daping Gui, Weishu Fan, Guangfen Cui, Wenjie Jia, Andan Zhu, Jihua Wang

**Affiliations:** ^1^Flower Research Institute, Yunnan Academy of Agricultural Sciences, National Engineering Research Center for Ornamental Horticulture, Kunming, China; ^2^Joint Lab of Yunnan Seed Industry, Kunming, China; ^3^Germplasm Bank of Wild Species, Kunming Institute of Botany, Chinese Academy of Sciences, Kunming, China

**Keywords:** *Lilium* species, lily cultivars, plastid genome, phylogenetic relationship, maternal origin

## Abstract

Lilies are one of the most important ornamental flowers worldwide with approximately 100 wild species and numerous cultivars, but the phylogenetic relationships among wild species and their contributions to these cultivars are poorly resolved. We collected the major *Lilium* species and cultivars and assembled their plastome sequences. Our phylogenetic reconstruction using 114 plastid genomes, including 70 wild species representing all sections and 42 cultivars representing six hybrid divisions and two outgroups, uncovered well-supported genetic relationships within *Lilium*. The wild species were separated into two distinct groups (groups A and B) associated with geographical distribution, which further diversified into eight different clades that were phylogenetically well supported. Additional support was provided by the distributions of indels and single-nucleotide variants, which were consistent with the topology. The species of sections *Archelirion, Sinomartagon* III, and *Leucolirion* 6a and 6b were the maternal donors for Oriental hybrids, Asiatic hybrids, Trumpet hybrids, and Longiflorum hybrids, respectively. The maternal donors of the OT hybrids originated from the two sections *Archelirion* and *Leucolirion* 6a, and LA hybrids were derived from the two sections *Leucolirion* 6b and *Sinomartagon*. Our study provides an important basis for clarifying the infrageneric classification and the maternal origin of cultivars in *Lilium*.

## Introduction

Lilies, belonging to the genus *Lilium* L., are one of the most important ornamental flowers worldwide and are used as cut flowers and potted and garden plants (Van Tuyl et al., 2011; Miller, 2017; Madhavan et al., 2021). There are about 100 wild species and thousands of cultivars in this genus (Tang et al., 2021). These wild species are widely distributed in the Northern Hemisphere and are mainly centered in Asia, North America, and Europe (Liang, 1995; McRae, 1998; Liang and Tamura, 2000; Patterson and Givnish, 2002). Various species are used in developing lily cultivars due to their valuable genetic diversity and good cross-compatibility (McRae, 1998). However, both the overall phylogeny of wild species and the origin of cultivars remain incompletely resolved.

The infrageneric classification of the genus *Lilium* has been a controversial issue since its establishment by Linnaeus in 1753, and its taxonomic systems have been modified repeatedly (Reichenbach, 1830; Baker, 1871; Wilson, 1925; Comber, 1949; De Jong, 1974; Liang, 1980; Haw and Liang, 1986; Nishikawa et al., 1999; Du et al., 2014a; Kim et al., 2019). Among them, Comber (1949) classified this genus into seven sections based on 13 morphological characteristics and two germination types, namely, *Martagon, Pseudolirium, Liriotypus, Archelirion, Sinomartagon, Leucolirion*, and *Daurolirion*, which was widely accepted, but there were still disputes about the definition of some sections and the division of several species. De Jong (1974) revised Comber's classification by incorporating *Dautolirion* into *Sinomartagon*, as well as separating campaniform-flowered species from *Sinomartagon* as a new section *Oxypetalum*. That means *Lilium* was divided into the sections *Martagon, Pseudolirium, Lilium* (*Liriotypus*), *Archelirion, Sinomartagon, Leucolirion*, and *Oxypetalum*, which was recognized to be more reasonable (Van Tuyl et al., 2018). However, phylogenetic analyses based on molecular approaches have revealed that most of the sections are not monophyletic, and some species with similar morphological characteristics are distantly related (Mitchell, 1998; Nishikawa et al., 1999, 2001; Hayashi and Kawano, 2000; Lee et al., 2011; Du et al., 2014a). Therefore, the reevaluation of the classification of the genus *Lilium* is necessary.

Lily cultivars are bred mainly by interspecific or intersectional hybridization among hybrids and/or species (Van Tuyl et al., 2018) and have been classified into nine different divisions by the Royal Horticultural Society according to parentage and particular characteristics: Asiatic hybrids (A), Martagon hybrids, Euro-Caucasian hybrids, American hybrids, Longiflorum lilies (L), Trumpet and Aurelian hybrids (T), Oriental hybrids (O), Other hybrids [e.g., the Oriental × Trumpet hybrids (OT) and the Longiflorum × Asiatic hybrids (LA)], and all species and their varieties and forms (https://www.rhs.org.uk/plants/plantsmanship/plant-registration/lily-cultivar-registration/lily). The cultivars of the A, O, L, T, OT, and LA hybrids dominate the current market (Lim and Van Tuyl, 2007; Du et al., 2019). According to the division descriptions in the Royal Horticultural Society's International Lily Register, the A hybrids are derived from the hybridization of wild species within section *Sinomartagon*, including *L. amabile, L. bulbiferum, L. callosum, L. cernuum, L. concolor, L. dauricum, L. davidii, L. lancifolium, L. lankongense, L. leichtlinii, L. maculatum, L. pumilum, L. wardii*, and *L. wilsonii*; the O hybrids are derived from section *Archelirion*, including *L. auratum, L. japonicum, L. nobilissimum, L. rubellum*, and *L. speciosum*; the T hybrids are derived from subsection *Leucolirion* 6a, including *L. brownii, L. henryi, L. leucanthum, L. regale, L. rosthornii, L. sargentiae*, and *L. sulphureum*; and the L hybrids are derived from subsection *Leucolirion* 6b, including *L. formosanum, L. longiflorum, L. philippinense*, and *L. wallichianum* (Van Tuyl et al., 2011, 2018; Hoshino et al., 2018). However, the specific wild species that contributed to the breeding of lilies have not been confirmed by genomic analysis.

Molecular approaches have been widely used in the phylogenetic analysis of *Lilium* (Nishikawa et al., 1999, 2001; Hayashi and Kawano, 2000; Patterson and Givnish, 2002; Gao et al., 2013; Du et al., 2014a; Dierckxsens et al., 2017). Nishikawa et al. (1999, 2001); Huang et al. (2018) evaluated the phylogenetic relationships of *Lilium* based on ITS sequences and found that section *Daurolirion* was not independent of section *Sinomartagon, L. henryi* and *L. bulbiferum* should be classified into subsection 6a and *Sinomartagon*, respectively, and *Sinomartagon* was polyphyletic and divided into five clades. Du et al. (2014a) focused on *Sinomartagon* 5c and suggested that subsection 5c should be classified into the true subsection 5c and the section *Lophophorum*. Gao et al. (2013, 2015) investigated the phylogenetic and biogeographic characteristics, divergence times, and diversification rates of the genus *Lilium*, and the results confirmed that sections of *Lilium* are paraphyletic and that *Nomocharis* is nested within *Lilium*. Previous studies have resolved some controversies in the classification of *Lilium*, but as Kim et al. (2019) pointed out, there are still low supporting values and unresolved branches in the phylogeny of *Lilium*. Plastid genomes (plastomes) can provide more detailed information compared to single-marker approaches and have been widely used to clarify the phylogenetic relationships in plants, particularly in some groups with diverse morphological traits and complex evolutionary histories (Hajibabaei et al., 2007; Pfenninger et al., 2007; Zhang et al., 2017, 2019; Rabah et al., 2019; Valcárcel and Wen, 2019; Wang et al., 2020), as well as to resolve the maternal ancestors of various cultivars (Nikiforova et al., 2013; Carbonell-Caballero et al., 2015; Viljoen et al., 2018; Wen et al., 2020). Complete plastomes have already been used in the systematic studies of *Lilium*, and the level of statistical support for the branches observed has been very high (Du et al., 2017; Kim et al., 2017, 2019; Li et al., 2021). However, one common shortcoming of previous studies on *Lilium* is the insufficient number of taxa sampled. Increasing the taxon sampling is a widely accepted approach to improving phylogenetic accuracy (Zwickl and Hillis, 2002; Heath et al., 2008; Kim et al., 2019).

In this study, 80 new plastomes in *Lilium* were obtained through next-generation sequencing (NGS), and 34 plastomes were obtained from GenBank, which included 70 wild taxa covering all sections (i.e., *Martagon, Pseudolirium, Archelirion, Leucolirion, Lilium, Sinomartagon*, and *Daurolirion*) (Comber, 1949) and 42 cultivars covering the current mainstream hybrids on the market (i.e., Oriental hybrids, Asiatic hybrids, Trumpet hybrids, Longiflorum hybrids, OT hybrids, and LA hybrids), with *Fritillaria karelinii* and *Hosta yingeri* as outgroups (Supplementary Table 1). A total of 114 plastomes were analyzed to (1) clarify the phylogenetic relationships among sections in the genus *Lilium* and (2) elucidate the genetic contributions of wild species to the cultivars.

## Materials and Methods

### Plant Materials

The plant materials sequenced in this study were collected from the lily germplasm bank of the Flower Research Institute, Yunnan Academy of Agricultural Sciences, and the Germplasm Bank of Wild Species, Kunming Institute of Botany, Chinese Academy of Sciences, and comprised a total of 80 lily plants.

### Total DNA Extraction, Sequencing, Assembly, and Annotation

Fresh leaves from the 80 lily plants were collected and quickly frozen in liquid nitrogen for DNA extraction. Total genomic DNA was extracted using the modified cetyltrimethylammonium bromide protocol (Doyle and Doyle, 1987) and used for library construction with the Illumina TruSeq Nano DNA Library Prep Kit. The libraries were sequenced on the Illumina NovaSeq 5000 platform at Biomarker Technologies Co., Ltd (Beijing, China), generating ~6 Gbp of paired-end data (2 × 150 bp) per sample.

Raw data were evaluated by FastQC (Leggett et al., 2013) and low-quality data were trimmed using Trimmomatic v0.36 (Bolger et al., 2014) with default parameters. All the Illumina data were *de novo* assembled with NOVOPlasty v2.7.2 (Dierckxsens et al., 2017) in “chloro” mode, using *atpA* as a seed sequence and the plastome of *L. henryi* (NC_035570.1) as a reference. The raw data reported herein are available in the National Genomics Data Center (NGDC) Genome Sequence Archive (GSA) (https://bigd.big.ac.cn/gsa/) under the accession number CRA005744.

The plastomes were annotated using PGA (Qu et al., 2019) based on homology to *L. henryi* plastid genes. The exact gene and intron boundaries and any missing annotations were manually checked and edited with Geneious 7.1.4 (Kearse et al., 2012) if needed. The assembled plastomes were submitted to Genome Warehouse under BioProject PRJCA007716. All taxon sampling experiments and data information are listed in Supplementary Table 1, and the statistics of the plastome assemblies are summarized in Supplementary Table 2. Additionally, 32 plastomes of the genus *Lilium* and two outgroups (*F. karelinii* and *H. yingeri*) from GenBank were selected for inclusion in this study. These public data were examined carefully and those plastomes with incorrect assembly or potentially inaccurate species identification were excluded for analyses, and only one representative plastome of the same species was used considering the large data quantity.

### Sequence Alignments and Phylogenetic Analyses

A phylogenetic tree was constructed from 70 complete plastome sequences of wild species to clarify the phylogenetic relationships of *Lilium*. Additionally, a phylogenetic tree was constructed from 112 plastid sequences (including 70 *Lilium* plastomes of wild species and 42 cultivars) to clarify the relationships between wild species and cultivars.

All whole plastomes have a typical quadripartite structure, including a large single-copy (LSC) region, a small single-copy (SSC) region, and a pair of inverted repeat (IR) regions. The LSC, SSC, and one IR region were aligned with MAFFT v7.407 separately (Katoh and Standley, 2013), the low-quality aligned regions were removed by Gblocks (Dereeper et al., 2008) with the parameters –t = d and –b5 = h, and then the trimmed sequences were concatenated by FASconCAT V1.0.pl. Phylogenetic analyses based on complete plastome sequences (LSC + SSC + IR) were estimated with maximum likelihood (ML), Bayesian inference (BI) (Bouckaert et al., 2014), and maximum parsimony (MP) methods. ML analysis was constructed using RAxML v8.2.12 with the GTRGAMMAI substitution model (Stamatakis, 2014), which was determined as the best-fitting model by jModelTest2.0 (Darriba et al., 2012), with 100 bootstrap replicates. BI was performed using MrBayes v3.2 (Ronquist et al., 2012) with a default of two runs, four chains, and unlinked rates for two million generations, with sampling every 2,000 generations. The burn-in was set to discard the first 25% of the trees. A majority-rule consensus tree of all the remaining trees was used to calculate Bayesian posterior probability (BPP) values. MP analysis was performed using MEGA v7.0 (Kumar et al., 2016) with 100 bootstrap replicates and the default parameters.

### Single-Nucleotide Variation and Indel (Insertion and Deletion) Analysis

The plastomes of the wild species were pairwise aligned with *L. canadense* using MAFFT v7.407 (Katoh and Standley, 2013), generating 69 plastome pairs for further SNV and indel analysis. The SNVs and indels were called by comparing each nucleotide status to the reference *L. canadense*. The coordinate positions of nucleotide substitutions and gaps in the alignments were scanned and extracted.

## Results

### Genome Structure and Organization of Lily Plastomes

**Figure 1:**
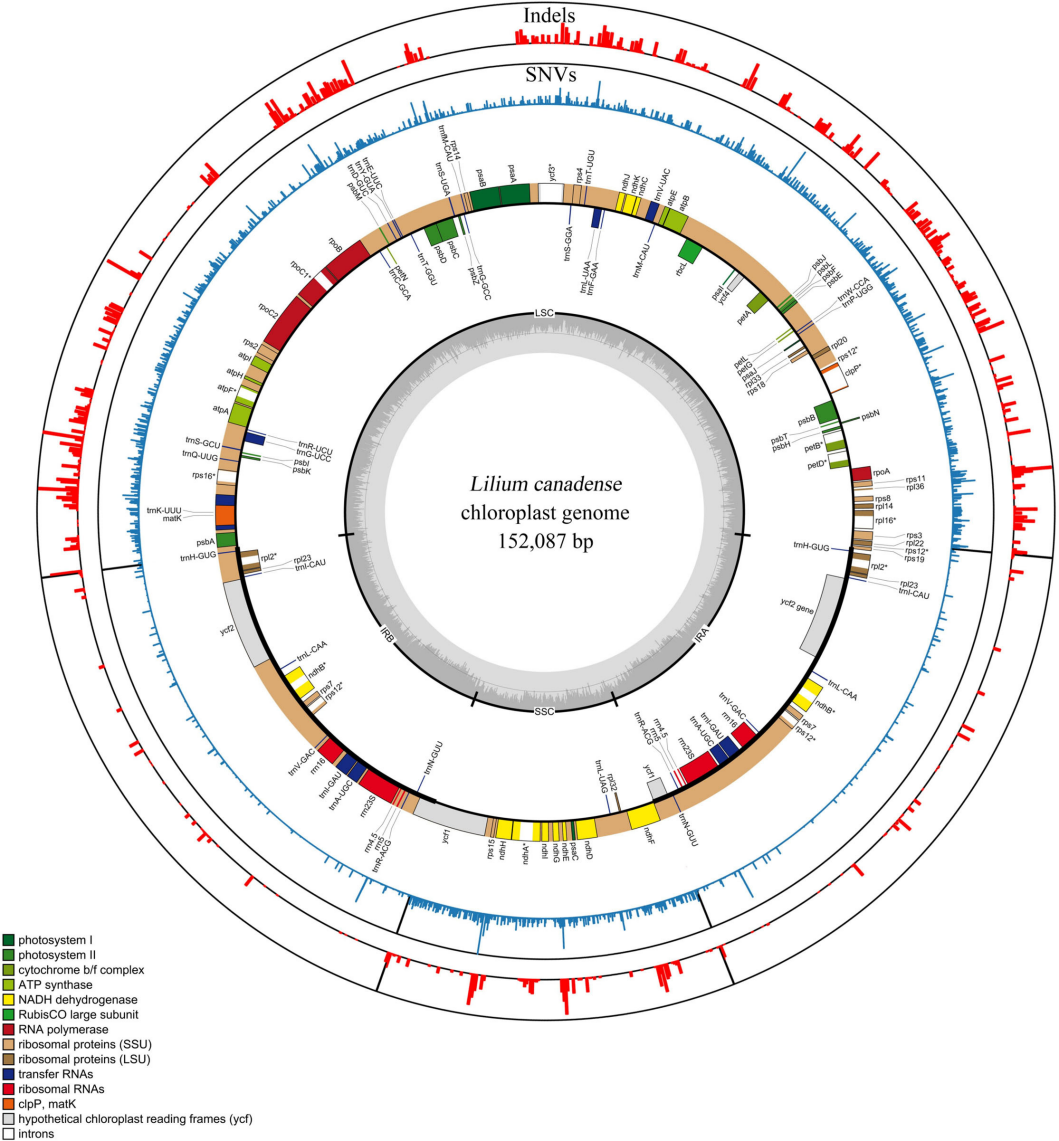
Genomic map and variations of the genus *Lilium* represented over a circular map of the *Lilium canadense* plastome. SNVs and indels are shown in red and blue in the outer layers, and the genomic map is shown in the inner layer. The frequency of the SNVs and indels is represented by the height of the colored bars. Genes shown on the outside of the genomic map are transcribed clockwise and those inside are transcribed counterclockwise. Gene functional groups are color-coded.

The plastomes of 80 *Lilium* taxa were successfully assembled into a single circular molecule. The full-length plastome varied between 151,802 and 153,194 bp, with Med = 152,623 bp and $\bar{x}$ = 152,574 ± 345 bp. The genome architecture of all plastomes was a typical quadripartite circular molecule found in most photosynthetic angiosperms, including a large single-copy (LSC) region of 81,224–82,571 bp (Med = 82,045 bp and $\bar{x}$ = 81, 994 ± 329 bp), and a small single-copy (SSC) region of 17,343–17,656 bp (Med = 17,531 bp and $\bar{x}$ = 17, 540 ± 70 bp), which were separated by a pair of inverted repeat (IR) regions of 26,394–26,624 bp (Med = 26,519 bp and $\bar{x}$ = 26,520 ± 44 bp). The total GC content of all plastomes was nearly identical (37.0–37.1%) (Figure 1; Supplementary Table 2).

The *Lilium* plastomes comprised a total of 130 genes (112 unique genes), including 84 protein-coding genes (78 unique), 38 tRNA genes (30 unique), and eight rRNA genes (four unique). Among the detected genes, 18 unique genes contained introns, of which 15 genes (*atpF, ndhA, ndhB, petB, petD, rpl16, rpoC1, rps16, rpl2, trnG-UCC, trnK-UUU, trnL-UAA, trnV-UAC, trnA-UGC, and trnI-GAU*) contained one intron, and three genes (*clpP, ycf3*, and *rps12*) had two introns (Figure 1; Supplementary Table 3). The structure, gene arrangement, and content of the plastomes in *Lilium* exhibited a high degree of conservation and were basically consistent with the characteristics of plastomes found in other genera (Bayly et al., 2013; Carbonell-Caballero et al., 2015; Zhao et al., 2015).

### Phylogenetic Relationships of the Wild Species in *Lilium*

**Figure 2:**
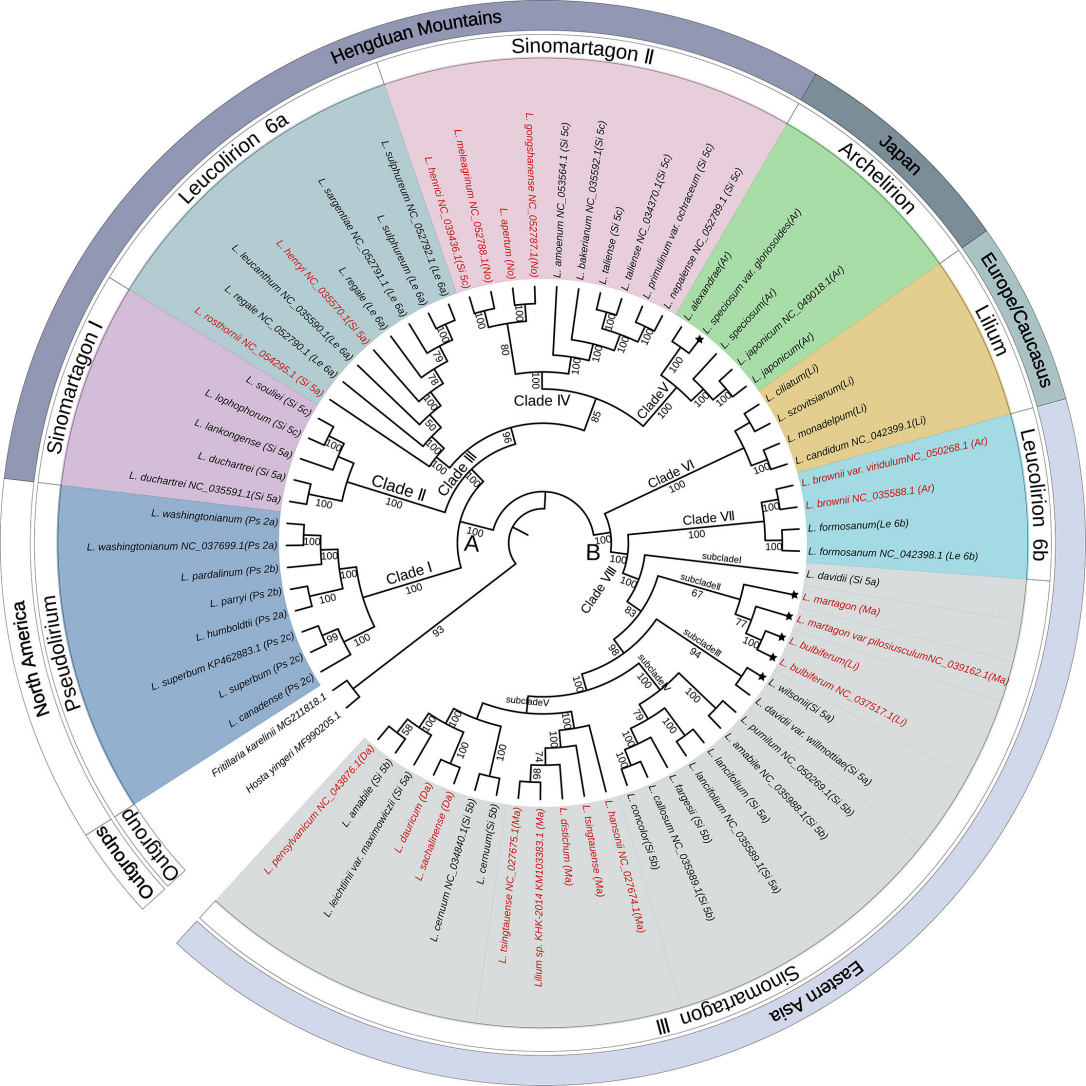
Phylogenetic tree constructed from the plastome sequences (LSC + IR + SSC) of 70 *Lilium* taxa and two outgroup taxa using the maximum likelihood method. The outermost layer represents the general geographical distribution. The second circle of the outer layer represents the classification of the genus *Lilium*. The phylogenetic tree is shown in the inner layer. Numbers associated with the branches are bootstrap values. A and B indicate the two major groups distinguished based on all three phylogenetic reconstruction methods. Clades I, II, III, IV, V, VI, VII, and VIII indicate the major clades of *Lilium* species. Terminal names comprise species. Species that conform to Comber's classification are shown in black color and those showing inconsistent placements are shown in red color. The asterisk indicates that the geographical distribution of the species is inconsistent with the outermost layer. Species abbreviation in sections: Ps, *Pseudolirium*; Le, *Leucolirion*; Ar, *Archelirion*; Li, *Lilium*; Si, *Sinomartagon*; Ma, *Martagon*; Da, *Dautolirion*; and No, *Nomocharis*-like *Lilium*.

The complete plastome sequences of 70 wild species and two outgroups were used to perform phylogenetic analysis. The geographical distributions of the analyzed species were mapped to the phylogeny. The phylogenetic tree using ML, MP, and BI yielded identical topologies (Figure 2; Supplementary Figures S1, S2). Therefore, we utilized the ML tree for all subsequent results and discussions. The three approaches displayed two clearly separated groups (groups A and B) that further diversified into eight different clades supported with high bootstrap values (Figure 2). Group A comprised five clades (labeled as Clades I, II, III, IV, and V), which contained all North American species, Hengduan Mountain species, Japanese species, *L. rosthornii*, and *L. henryi* with 100% bootstrap support. Group B comprised three clades (Clades VI, VII, and VIII), which consisted of all European species and all East Asian species, except the Japanese species, and all the subclades were highly supported.

Within group A, all sampled species from section *Pseudolirium* were distributed in North America, including *L. humboldtii, L. washingtonianum, L. pardalinum, L. parryi, L. canadense*, and *L. superbum*, and constituted a monophyletic clade (Clade I) with robust support (BS = 100%). Clade II (*Sinomartagon* I) contained *L. duchartrei, L. lankongense, L. lophophorum*, and *L. souliei* from section *Sinomartagon* with 100% bootstrap support, which are endemic to China and distributed in the Hengduan Mountains (Liang, 1980). *Lilium duchartrei* and *L. lankongense*, which belonged to subsection *Sinomartagon* 5a Comber, have similar characteristics, including stoloniferous bulbs, scattered leaves, revolute tepals, and dark purple spots, on the flowers and were once considered to be a single species in earlier studies (Haw and Liang, 1986; Liang and Tamura, 2000). *Lilium lophophorum* and *L. souliei* with campanulate flowers were placed in *Sinomartagon* 5c by Comber (1949), but *L. lophophorum* was later adjusted into section *Oxypetalum* by De Jong (1974), both of which were classified into section *Lophophorum* in the Flora of China (Liang and Tamura, 2000). *Lilium rosthornii* and *L. henryi*, which were placed into *Sinomartagon* 5a by Comber (1949), were clustered with the species from *Leucolirion* 6a within Clade III with BS 100%. Clade IV (BS = 85%) included two clusters: one cluster was composed of *L. apertum, L. gongshanense, L. henricii*, and *L. meleagrinum*. *Lilium apertum, L. gongshanense*, and *L. meleagrinum* were once regarded as the species of the genus *Nomocharis*. Another cluster of Clade IV contained *L. amoenum, L. bakerianum, L. primulinum* var. *ochraceum, L. nepalense*, and *L. taliense*, which are only found in the Himalayas or Hengduan Mountains (Haw and Liang, 1986) and were placed into *Sinomartagon* 5c by Comber (1949). Clade V comprised only the Japanese species classified as section *Archelirion* with robust support (BS = 100%). *Lilium alexandrae, L. japonicum, L. speciosum*, and *L. speciosum* var. *gloriosoies* are generally characterized by very large petals, bowl to open funnel-shaped flowers, and broad, scattered leaves that are distinctly petiolate (Pelkonen and Pirttilä, 2012).

Within group B, three clades were recovered. All species from section *Lilium* analyzed in this study formed a monophyletic clade (Clade VI) with strong support (BS = 100%) and are native to Europe. Clade VII consisted of *L. formosanum, L. brownii*, and *L. brownii* var. *viridulum* with a bootstrap value of 100%, indicating that *L. brownii* were closely related to *Leucolirion* 6b. Clade VIII contained most species from *Sinomartagon* 5a and 5b and the species from sections *Daurolirion* and *Martagon* with BS 100%, which was further subdivided into five subclades. *Lilium davidii* from *Sinomartagon* 5a formed an independent lineage (subclade I), which was sister to the other subclades. Interestingly, *L. bulbiferum, L. martagon*, and *L. martagon* var. *pilosiusculum*, which are widely distributed in Europe, formed subclade II (BS = 67%) within *Sinomartagon* III. *Lilium bulbiferum* was classified into section *Lilium*, and *L. martagon* was considered as a species of section *Martagon* (Comber, 1949). Subclade III comprised *L. wilsonii* and *L. davidii* var. *willmottiae* from *Sinomartagon* 5a with high support (BS = 94%). Subclade IV (BS = 100%) included *L. pumilum, L. amabile, L. lancifolium, L. fargesii, L. callosum*, and *L. concolor*, all of which belonged to *Sinomartagon* 5b except *L. lancifolium* (*Sinomartagon* 5a). Subclade V was further subdivided into two clusters: one composed of *Martagon* species except for *L. martagon* and *L. martagon* var. *pilosiusculum* (BS = 100%), and the other containing *L. sachalinense, L. dauricum, L. pensylvanicum, L. cernuum, L. leichtlinii* var. *maximowiczii*, and *L. amabile*. *Lilium sachalinense* and *L. dauricum* are supposed to be synonyms for *L. pensylvanicum* in WCSP (https://wcsp.science.kew.org/), which was classified into section *Daurolirion* by Comber (1949), but De Jong (1974) placed it into section *Sinomartagon* 5a, which was later supported by molecular systematics (Nishikawa et al., 1999).

### Genomic Variations Among the Main Clades of Wild *Lilium* Species

**Figure 3:**
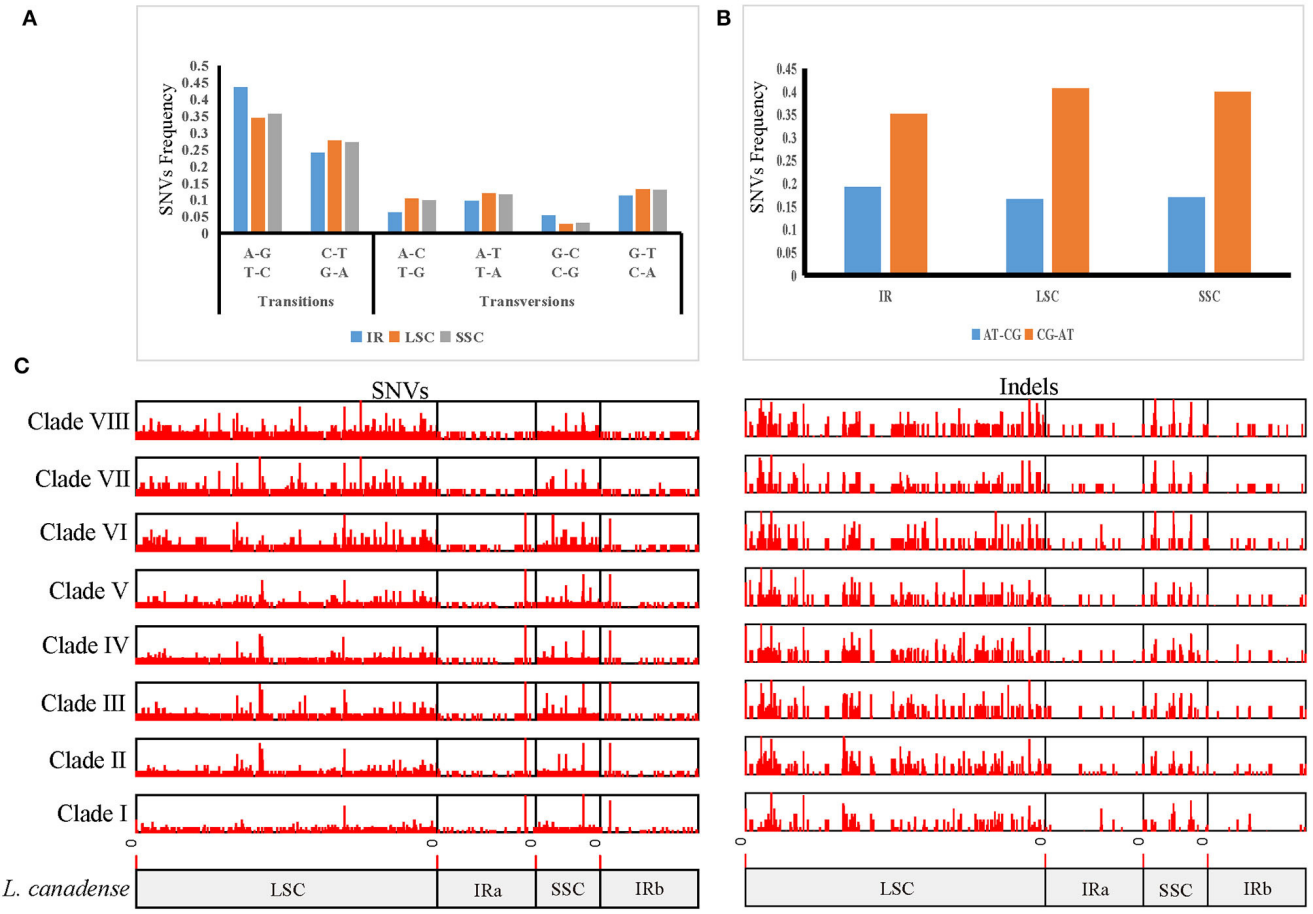
Mutation patterns in *Lilium* plastomes. **(A)** Mutation spectra of the IR, LSC, and SSC regions. **(B)** AT-biased mutations in the IR, LSC, and SSC regions. **(C)** Distribution of indels and SNVs along plastomes among eight *Lilium* clades. Mutations were found within each clade compared to *L. canadense*, which belongs to Clade I.

In order to clarify the variation in *Lilium*, we used the plastome sequences of *L. canadense*, a species of section *Pseudolirium* that is native to North America, as a reference, and identified 5,924 SNVs and 2,171 indels, totaling 8,095 mutations among the 70 plastomes in *Lilium*. The SNV mutations in *Lilium* were driven by an increased frequency of GC → AT transitions and showed an A/T bias, and the GC → AT transitions of the LSC region were higher than in the SSC and IR regions. The average variations were 39 SNVs per kb and 14.3 indels per kb. The most variable region was the SSC region with 83.2 mutations per kb, followed by the LSC region with 73.7 mutations per kb, which might represent hotspots for genetic variation (Figures 3A,B; Supplementary Table 4).

Based on the results of the phylogenetic analysis, each clade was labeled, and the frequencies of variations among clades were compared. Among these clades, the frequencies of variations ranged from 3.3 to 10.2 mutations per kb (0.7–1.8 indels per kb and 2.6–8.4 SNVs per kb). The minimum value was recorded for Clade I, which consisted of all species of section *Pseudolirium* distributed in North America, and the maximum value was recorded for Clade VI, which was composed of all species of section *Lilium* distributed in Europe. The results revealed that the frequency of indels was lower than that of SNVs in all clades; the SNVs and indels of clades (Clades VI, VII, and VIII) from group B were higher than those (Clades I, II, III, IV, and V) from group A, since group B was more distantly related to the reference species *L. canadense* which belonged to Clade I (Figure 3C; Supplementary Table 5).

### Maternal Contributions of Wild *Lilium* Species to Lily Cultivars

**Figure 4:**
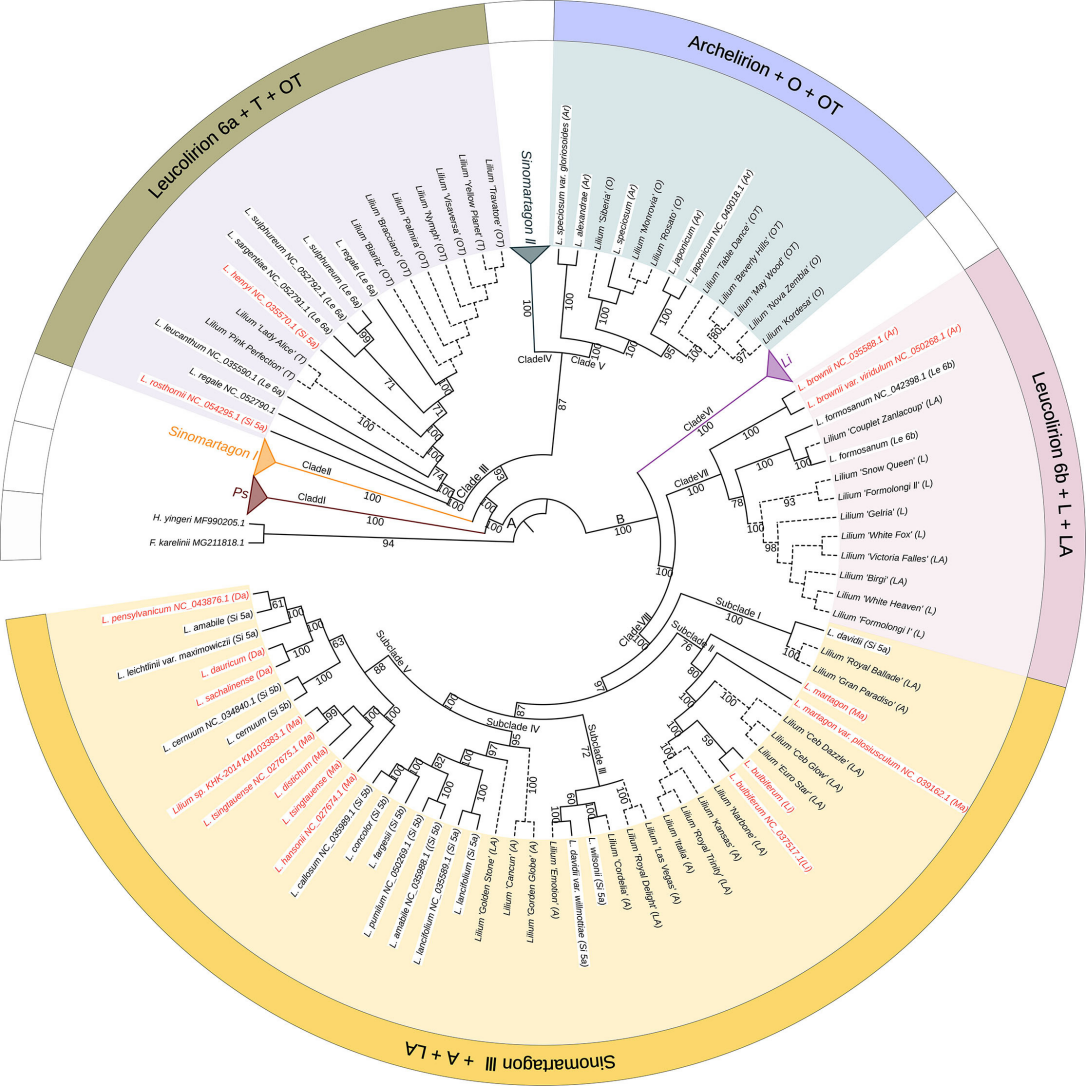
The phylogenetic relationships between wild species and cultivars in *Lilium* using plastomes based on the maximum likelihood method. Numbers associated with the branches are bootstrap values. A and B indicate the two major groups distinguished based on all three phylogenetic reconstruction methods. Clades I, II, III, IV, V, VI, VII, and VIII indicate the major clades of *Lilium* species. Terminal names comprise wild species and cultivars. Species that conform to Comber's classification are shown in black color and those inconsistent placements are shown in red color. Solid lines represent wild species, while dotted lines represent cultivars. Ps, *Pseudolirium*; Le, *Leucolirion*; Li, *Lilium*; Ar, *Archelirion*; Si, *Sinomartagon*; Ma, *Martagon*; Da, *Dautolirion*; A, Asiatic hybrids; O, Oriental hybrids; L, Longiflorum hybrids; T, Trumpet hybrids; OT, Oriental-Trumpet hybrids; LA, Longiflorum-Asiatic hybrids.

To elucidate the relationships between wild species and cultivated lilies and trace the maternal origins of the cultivars, we constructed a phylogeny of the most important *Lilium* species using 114 complete plastomes, including 72 wild accessions and 42 cultivars, rooted by *F. karelinii* and *H. yingeri* (Figure 4). The phylogenetic tree showed that the cultivars originating from different hybrids formed four separate clades with wild species in different sections of the genus *Lilium*.

All T cultivars, including *L*. ‘Pink Perfection', *L*. ‘Lady Alice', and *L*. ‘Yellow Planet', clustered with *Leucolirion* 6a species within Clade III. All O cultivars, including *L*. ‘Siberia', *L*. ‘Monrovia', *L*. ‘Rosato', *L*. ‘Nova Zembla', and *L*. ‘Kordesa', clustered with *Archelirion* species within Clade V. *Nine* OT cultivars were split into two parts, with six (*L*. ‘Biaritz', *L*. ‘Bracciano', *L*. ‘Palmira', *L*. ‘Nymph', *L*. ‘Visaversa', and *L*. ‘Travatore') clustering with *Leucolirion* 6a and three (*L*. ‘Table dance', *L*. ‘Beverly Hills', and *L*. ‘May Wood') clustering with *Archelirion*. All L cultivars, including *L*. ‘Snow Queen', *L*. ‘Formolongi II', *L*. ‘Gelria', *L*. ‘White Fox', *L*. ‘White Heaven', and *L*. ‘Formolongi I', clustered with *Leucolirion* 6b species within Clade VII. All A cultivars, including *L*. ‘Gran Paradiso', *L*. ‘Kansas', *L*. ‘Italia', *L*. ‘Las Vegas', *L*. ‘Emotion', *L*. ‘Cordelia', *L*. ‘Gorden Globe', and *L*. ‘Cancun', clustered with *Sinomartagon* III species within Clade VIII. Similar to OT, 11 LA cultivars were scattered in two clades, three (*L*. ‘Couplet Zanlacoup', *L*. ‘Victoria Falles', and *L*. ‘Birgi') clustered with *Leucolirion* 6b and eight (*L*. ‘Royal Ballade', *L*. ‘Ceb Dazzle', *L*. ‘Ceb Glow', *L*. ‘Euro Star', *L*. ‘Narbone', *L*. ‘Royal Trinity', *L*. ‘Royal Delight', and *L*. ‘Golden Stone') clustered with *Sinomartagon* III.

## Discussion

We comprehensively investigated the phylogenetic relationships between the wild species of *Lilium* and the maternal origin of cultivars by densely sampling *Lilium*. The complete plastomes provided two well-resolved phylogenetic trees of *Lilium* (Figures 2, 4), which have provided new and valuable information for resolving important controversies regarding the evolution of this genus and for tracing the species involved in the breeding of the cultivars.

### Phylogenetic Delimitation in the Genus *Lilium*

We obtained a highly resolved phylogenetic tree based on the complete plastomes of 70 wild *Lilium* species and two outgroups, providing strong, unambiguous support for the phylogenetic relationships of the wild lilies. The two distinct groups (groups A and B) that we recovered here are consistent with the results of Kim et al. (2019), who showed that the genus *Lilium* was divided into two major lineages composed of Asia + Europe species and Hengduan Mountains + North America species. In this study, the two groups were further diversified into eight distinct major clades, which is not completely consistent with previous studies wherein the genus *Lilium* was divided into seven sections based on the morphological analyses (Comber, 1949; De Jong, 1974). Sections *Pseudolirium, Archelirion*, and *Lilium* were recovered here; section *Sinomartagon* was shown to be polyphyletic with three distinct clades; sections *Martagon* and *Daurolirion* were nested in the section *Sinomartagon* III; and *Leucolirion* 6a and 6b were confirmed to be distantly related.

#### *Pseudolirium, Archelirion*, and *Lilium* Are Appropriate as Independent Sections

Section *Pseudolirium* consisted of all North America species, constituting ~21 species typically having whorled leaves, erect stems, and rhizomatous to stoloniferous bulbs (Comber, 1949; Lighty, 1968; De Jong, 1974). This section was previously considered a unique taxon due to its New World Distribution (Lighty, 1968). Phylogenetic analyses using ITS sequences suggested that this section was monophyletic (Nishikawa et al., 1999, 2001; Du et al., 2014a). In this study, the eight chloroplast genomes of *Pseudolirium* formed a monophyletic clade (Clade I in Figure 2), confirming that *Pseudolirium* is appropriately treated as a single section.

Section *Archelirion* included *L. alexandrae, L. auratum, L. brownii, L. japonicum, L. nobilissimum, L. rubellum*, and *L. speciosum* in Comber's classification (Comber, 1949). Most are found only in the Japanese islands, except for *L. brownii*, which is distributed in Southeast China (McRae, 1998; Du et al., 2014b). Morphologically, *L. brownii* has white and trumpet-shaped flowers, which is very similar to *L. formosanum* and *L. longiflorum* of *Leucolirion* 6b (Du et al., 2014b; Liu et al., 2019). Molecular phylogenetics previously revealed that *L. brownii* was more closely related to *Leucolirion* 6b than *Archelirion* (Dubouzet and Shinoda, 1999; Nishikawa et al., 2001; Lee et al., 2011; Gao et al., 2012; Du et al., 2014a), and Du et al. (2014b) proposed that *L. brownii* should be classified into *Leucolirion* 6b. In this study, *L. brownii* and *L. brownii* var. *viridulum* clustered with *L. formosanum* from *Leucolirion* 6b in Clade VII, and other *Archelirion* species formed a monophyletic clade (Clade V), which was consistent with Kim et al. (2019). Therefore, we suggest that *Archelirion*, with the exclusion of *L. brownii*, is appropriate as an independent section.

Section *Lilium* was composed of European species, except *L. martagon*, in Comber's classification (Comber, 1949). Interestingly, *L. bulbiferum*, a species widely distributed in Europe, was distinguished from this section (Nishikawa et al., 1999, 2001; Ikinci et al., 2006; Muratović et al., 2010; Lee et al., 2011). Morphologically, *L. bulbiferum* with upright flowers differs from other species with Turk's cap-shaped flowers in this section. Lighty (1968) supposed that *L. bulbiferum* was derived from *L. dauricum* of section *Daurolirion*. Based on ITS sequences, Nishikawa et al. (1999) suggested that *L. bulbiferum* and *L. dauricum* should be included in section *Sinomartagon*. Our results showed that *L. bulbiferum* was placed far away from section *Lilium* and formed a branch (subclade II) with *L. martagon* and *L. martagon* var. *pilosiusculum* within *Sinomartagon* III. Therefore, we believe that section *Lilium* excluding *L. bulbiferum* is appropriate as an independent section.

#### *Martagon* and *Daurolirion* Should Not Be Regarded as Independent Sections

Section *Martagon* contained five species in Comber's classification (Comber, 1949), including *L. tsingtauense, L. distichum, L. hansonii, L. martagon*, and *L. medeoloides*, which were considered to be primitive in the genus *Lilium* due to their morphological characteristics of hypogeal and delayed germination, whorled leaves, jointed scales, and heavy seeds (Lighty, 1968). An ITS phylogeny showed that *Martagon* was monophyletic and sister to section *Sinomartagon* (Nishikawa et al., 1999; Lee et al., 2011; Nikiforova et al., 2013; Du et al., 2014a). Phylogenetic analyses using complete plastome sequences showed that *Martagon* was derived from section *Sinomartagon* (Kim et al., 2019), which was confirmed by this study. In our study, *Martagon* species formed two subclades within *Sinomartagon* III, *L. martagon* and *L. martagon* var. *pilosiusculum* formed a subclade (subclade II) with *L. bulbiferum*, and other *Martagon* species clustered in subclade V. Additionally, both *Martagon* and *Sinomartagon* species are native to Eastern Asia, except for *L. martagon*, which is widely distributed in Eurasia (Liang and Tamura, 2000). These results implied that *Martagon* could not be regarded as an independent section.

Section *Daurolirion* was highly contentious in Comber's classification. According to the morphological characteristics of scattered leaves, articulate and white scales, and upright flowers, *L. dauricum* was regarded as a monotypic section by Comber (1949). However, it is generally believed that *Daurolirion* does not constitute a separate section (Nishikawa et al., 1999, 2001; Gao et al., 2013; Du et al., 2014a). Geographically, *L. dauricum* is distributed in Eastern Asia, as are most species of *Sinomartagon* 5a and 5b, and they all hybridize well (McRae, 1998). De Jong (1974) adjusted this section into *Sinomartagon* 5a. Phylogenies based on ITS or plastome sequences showed that *Daurolirion* formed a clade within *Sinomartagon* (Nishikawa et al., 1999, 2001; Gao et al., 2013; Du et al., 2014a; Kim et al., 2019). Our results showed that *L.dauricum* was placed in *Sinomartagon* III, which provided further support for the claim that *Daurolirion* is not an independent section.

#### *Sinomartagon* Is Polyphyletic and Should Be Redefined

Comber (1949) classified more than 30 species distributed in China into section *Sinomartagon* and further divided them into three subsections, namely, 5a, 5b, and 5c. This section, especially 5c, is the most complicated and controversial section in the infrageneric classification of the genus *Lilium*. De Jong (1974) separated some species with campanulate flowers from subsection 5c as a separate section named *Oxypetalum*. Subsection 5c was considered as a section *Lophophorum* in the Flora of China (Liang and Tamura, 2000). A molecular phylogeny based on ITS sequences also indicated that *Sinomartagon* was polyphyletic (Nishikawa et al., 1999, 2001; Du et al., 2014a). Nishikawa et al. (2001) considered that *Sinomartagon* should be divided into four groups, with 5a and 5b constituting the true section *Sinomartagon*, while Du et al. (2014a) suggested that subsection 5c should be classified into the true 5c and section *Lophophorum*.

In this study, *Sinomartagon* was divided into three major clades: Clade II, IV, and VIII. None of the clades showed sister relationships with the other clades. Clade II, which contained *L. duchartrei, L. lankongense, L. lophophorum*, and *L. souliei* belonging to different subsections, has not been recovered in previous works. *Lilium duchartrei* and *L. lankongense* with revolute tepals were classified into 5a, while *L. lophophorum* and *L. souliei* with campanulate flowers were divided into 5c and were considered as section *Lophophorum* in the Flora of China (Comber, 1949; De Jong, 1974; Haw and Liang, 1986; Liang and Tamura, 2000). Although these species have different morphological characteristics, they are endemic to China and are distributed in the Hengduan Mountains (Liang, 1980). Our results strongly supported that this clade was separate from the remaining species of *Sinomartagon*, and thus it should possibly be considered as a separate section. Clade IV contained many rare species, the classification of which is confusing and inconsistent. *Lilium henrici* and *L. amoenum* were classified into 5c by Comber (1949) and then separated into section *Oxypetalum* by De Jong (1974). *Lilium apertum, L. gongshanense*, and *L. meleagrinum* were considered as *Nomocharis*-like *Lilium* species and once belonged to the genus *Nomocharis* (Haw and Liang, 1986). It is generally believed that *Nomocharis* was closely related to *Sinomartagon* 5c, which was confirmed by our results. Clade VIII was complicated by the inclusion of sections *Martagon, Daurolirion*, and *Sinomartagon* 5a and 5b. As discussed above, these species from different sections mostly co-occur within Eastern Asia and have a close relationship, as supported by this study. Our results showed that *Sinomartagon* is polyphyletic, including many distantly related species. Therefore, we suggested that *Sinomartagon* should be redefined.

#### *Leucolirion* 6a and 6b Should Be Considered as Two Independent Sections Rather Than Two Subsections

*Lilium henryi* and *L. rosthornii* have similar morphological characteristics, including orange reflexed flowers, prominent papillae, and pubescent nectaries, and were previously placed into *Sinomartagon* 5a by Comber (Comber, 1949; Du et al., 2014a). However, *L. henryi* hybridizes well with subsection 6a and is one of the parents of “Aurelian hybrids” (McRae, 1998). Phylogenetic analysis based on ITS sequences also showed that *L. henryi* was closely related to subsection 6a (Nishikawa et al., 1999; Du et al., 2014a). *Lilium brownii* was classified into *Archelirion* by Comber (Comber, 1949), but it was divided into *Leucolirion* in the Flora of China (Liang and Tamura, 2000). Based on ITS sequences, Du et al. (2014a) proposed that *L. henryi* and *L. rosthornii* should be placed into 6a, and *L. brownii* should be placed into 6b, which was further supported by our results. As can be seen from Figure 2, *L. henryi* and *L. rosthornii* formed a clade (Clade III) with *Leucolirion* 6a, while *L. brownii* formed another clade (Clade VII) with 6b. These two clades were distantly separated with strong support and were scattered among groups A and B, respectively, which is congruent with previous phylogenetic studies (Nishikawa et al., 1999, 2001; Du et al., 2014a; Kim et al., 2019). Therefore, we suggested that 6a and 6b should be considered as two independent sections rather than two subsections.

### Potential Maternal Origin of Modern Lily Cultivars

Many excellent horticultural traits and disease resistance characteristics exist in species belonging to different sections of *Lilium* (Lim et al., 2008). Therefore, interspecific or intersectional hybridization is one of the most important methods for developing new cultivars and improving the agronomical characteristics of lilies. The plastid phylogeny recovered here showed that the cultivars of different hybrids were distributed in four clades and clustered with wild species from different sections within *Lilium*. This confirmed that diverse maternal donors exist in cultivated lilies, indicating that modern lily cultivars may have originated from complex hybridization events involving multiple species.

#### Section *Sinomartagon* and the Maternal Origin of the A Hybrids

The A hybrids are common lily hybrids and present a wide variety of colors, including white, yellow, orange, pink, and red (Van Tuyl et al., 2018). A previous study on the origin of the A hybrids showed that they were derived from the hybridization of at least 11 species within section *Sinomartagon*, these being *L. davidii, L. concolor, L. cernuum, L. dauricum, L. pumilum, L. amabile, L. leichtlinii, L. lancifolium, L. duchartrei, L. lankongense*, and *L. bulbiferum* (Van Tuyl et al., 2011). Our sampling included all these potential wild parental species of A hybrids. Our results showed that all A cultivars were placed only within *Sinomartagon* III but were grouped with different wild species, indicating that the A hybrids were derived from *Sinomartagon* III and had multiple maternal donors. The most likely maternal ancestors were *L. davidii, L. davidii* var. *willmottiae, L. martagon, L. martagon* var. *pilosiusculum, L. bulbiferum, L. wilsonii, L. lancifolium, L. amabile, L. pumilum, L. fargesii, L. concolor*, and *L. callosum*.

#### Section *Archelirion* and the Maternal Origin of the O Hybrids

The O hybrids are the most commercial and important lilies due to their big, showy, and fragrant flowers (Van Tuyl et al., 2018) and were derived from hybridization within section *Archelirion*, including *L. auratum, L. japonicum, L. nobilissimum, L. rubellum***, **and *L. speciosum* (Van Tuyl et al., 2011). In this phylogeny, all O cultivars were nested in *Archelirion*, and *L. speciosum* and *L. japonicum* appeared to be the species that were most closely linked to these cultivars. Hence, *L. speciosum* and *L. japonicum* were likely the ovule donors of these cultivars. However, this speculation needs further research because the taxon sampling in this study did not completely cover section *Archelirion*.

#### Section *Leucolirion* 6a and the Maternal Origin of the T Hybrids

According to a previous report, the Trumpet hybrids were the result of interspecific hybridization within *Leucolirion* 6a involving *L. brownii, L. henryi, L. leucanthum, L. regale, L. rosthornii, L. sargentiae*, and *L. sulphureum* (Van Tuyl et al., 2018). The plastid phylogeny recovered here showed that all T cultivars formed a sister relationship with species within *Leucolirion* 6a. As our sampling included all potential wild parental species, our results suggested that the most likely maternal ancestors of T hybrids were *L. regale, L. sargentiae, L. sulphureum, L. rosthornii, L. leucanthum*, and *L. henryi*, but not *L. brownii*.

#### Section *Leucolirion* 6b and the Maternal Origin of the L Lilies

A previous study on the origin of L lilies showed that they derived from hybridization between *L. formosanum* and *L. longiflorum* (Van Tuyl et al., 2018). In this phylogeny, all L cultivars clustered with *L. formosanum* within *Leucolirion* 6b, indicating that *L. formosanum* was likely the maternal ancestor. However, *L. longiflorum* was not sampled in this study, and therefore this speculation needs further evaluation.

#### Maternal Origin of LA and OT Hybrids

Previous studies showed that LA hybrids were the result of crosses of *L. longiflorum* × A cultivars, while OT hybrids were the result of the hybridization of O × T (Van Creij et al., 1990; Van Tuyl et al., 1991). The plastid phylogeny recovered here showed that LA cultivars were scattered in two clades and clustered with species of *Leucolirion* 6b and *Sinomartagon*, respectively, indicating that both *Leucolirion* 6b and *Sinomartagon* contributed to the LA hybrids. As with the LA hybrids, the OT cultivars were divided into two parts and clustered with species of *Archelirion* and *Leucolirion* 6a, separately, revealing that *Archelirion* and *Leucolirion* 6a were the likely maternal donors of OT hybrids.

## Data Availability Statement

All the raw sequencing reads generated in this study were deposited in GSA database under the accession CRA005744 (https://ngdc.cncb.ac.cn/gsa/browse/CRA005744). All the assembled plastomes were submitted to GWH under BioProject PRJCA007716 (https://ngdc.cncb.ac.cn/search/?dbId=gwh&q=PRJCA007716).

## Author Contributions

JW and AZ conceived and designed the research. QD, DG, GC, and WJ collected and provided plant material. QD, FL, WF, and AZ analyzed the data. QD, AZ, and FL wrote the paper. QD and FL contributed equally to this work. All authors contributed to the article and approved the submitted version.

## Funding

This research was financially supported by the National Key Research and Development Program of China (2020YFD1000400) (to JW), the Major Science and Technology Project of Yunnan Provincial Department of Science and Technology (2019ZG006) (to JW), and the CAS Pioneer Hundred Talents Program (to AZ).

## Conflict of Interest

The authors declare that the research was conducted in the absence of any commercial or financial relationships that could be construed as a potential conflict of interest.

## Publisher's Note

All claims expressed in this article are solely those of the authors and do not necessarily represent those of their affiliated organizations, or those of the publisher, the editors and the reviewers. Any product that may be evaluated in this article, or claim that may be made by its manufacturer, is not guaranteed or endorsed by the publisher.

## Supplementary Material

Supplementary Figure S1 Phylogenetic tree constructed from the plastome sequences (LSC + IR + SSC) of 70 *Lilium* taxa and two outgroup taxa using the maximum parsimony method. The outer circle represents the classification of the genus *Lilium*. The phylogenetic tree is shown in the inner layer. Numbers associated with the branches are bootstrap values. A and B indicate the two major groups distinguished based on all three phylogenetic reconstruction methods. Clades I, II, III, IV, V, VI, VII, and VIII indicate the major clades of *Lilium* species. Terminal names comprise species. Species that conform to Comber's classification are shown in black color and those showing inconsistent placements are shown in red color. Species abbreviation in sections: Ps, *Pseudolirium*; Le, *Leucolirion*; Ar, *Archelirion*; Li, *Lilium*; Si, *Sinomartagon*; Ma, *Martagon*; Da, *Dautolirion*; and No, *Nomocharis*-like *Lilium*.

Supplementary Figure S2 Phylogenetic tree constructed from the plastome sequences (LSC + IR + SSC) of 70 *Lilium* taxa and two outgroup taxa using the Bayesian inference method. The outer circle represents the classification of the genus *Lilium*. The phylogenetic tree is shown in the inner layer. Numbers associated with the branches are bootstrap values. A and B indicate the two major groups distinguished based on all three phylogenetic reconstruction methods. Clades I, II, III, IV, V, VI, VII, and VIII indicate the major clades of *Lilium* species. Terminal names comprise species. Species that conform to Comber's classification are shown in black color and those showing inconsistent placements are shown in red color. Species abbreviation in sections: Ps, *Pseudolirium*; Le, *Leucolirion*; Ar, *Archelirion*; Li, *Lilium*; Si, *Sinomartagon*; Ma, *Martagon*; Da, *Dautolirion*; and No, *Nomocharis*-like *Lilium*.

Supplementary Table 1 The sample information including classification, distribution details, vouchers, sampling, and data source.

Supplementary Table 2 Sequencing data information and assembly statistics of plastomes.

Supplementary Table 3 Gene composition of the plastomes in *Lilium*.

Supplementary Table 4 Mutations in different regions of plastomes in *Lilium*.

Supplementary Table 5 Variant analysis among clades in *Lilium*.

## References

[B1] Baker J.. (1871). A new synopsis of all the known lilies. Gard. Chron. 104, 1650.

[B2] Bayly M. J. Rigault P. Spokevicius A. Ladiges P. Y. Ades P. K. Anderson C.. (2013). Chloroplast genome analysis of Australian eucalypts–Eucalyptus, Corymbia, Angophora, Allosyncarpia and Stockwellia (Myrtaceae). Mol. Phylogenet. Evol. 69, 704–716. 10.1016/j.ympev.2013.07.006 23876290

[B3] Bolger A. M. Lohse M. Usadel B. (2014). Trimmomatic: a flexible trimmer for Illumina sequence data. Bioinformatics 30, 2114–2120. 10.1093/bioinformatics/btu170 24695404 PMC4103590

[B4] Bouckaert R. Heled J. Kühnert D. Vaughan T. Wu C. H. Xie D.. (2014). BEAST 2: a software platform for Bayesian evolutionary analysis. PLoS Comput. Biol. 10, e1003537. 10.1371/journal.pcbi.1003537 24722319 PMC3985171

[B5] Carbonell-Caballero J. Alonso R. Ibanez V. Terol J. Talon M. Dopazo J. (2015). A phylogenetic analysis of 34 chloroplast genomes elucidates the relationships between wild and domestic species within the genus citrus. Mol. Biol. Evol. 32, 2015–2035. 10.1093/molbev/msv082 25873589 PMC4833069

[B6] Comber H. F.. (1949). A New Classification of the Genus Lilium. Lily Year Book of RHS (London: Springer), 86–105.

[B7] Darriba D. Taboada G. L. Doallo R. Posada D. (2012). jModelTest 2: more models, new heuristics and parallel computing. Nat. Methods 9, 772–772. 10.1038/nmeth.2109 22847109 PMC4594756

[B8] De Jong P.. (1974). Some notes on the evolution of lilies. N. Am. Lily Yearb. 27, 23–28.

[B9] Dereeper A. Guignon V. Blanc G. Audic S. Buffet S. Chevenet F.. (2008). Phylogeny. fr: robust phylogenetic analysis for the non-specialist. Nucleic Acids Res. 36(Suppl_2), W465–W469. 10.1093/nar/gkn180 18424797 PMC2447785

[B10] Dierckxsens N. Mardulyn P. Smits G. (2017). NOVOPlasty: de novo assembly of organelle genomes from whole genome data. Nucleic Acids Res. 45, e18. 10.1093/nar/gkw955 28204566 PMC5389512

[B11] Doyle J. J. Doyle J. L. (1987). A rapid DNA isolation procedure for small quantities of fresh leaf tissue. Phytochem. Bull. 19, 11–15.

[B12] Du F. Wang T. Fan J. M. Liu Z. Z. Zong J. X. Fan W. X.. (2019). Volatile composition and classification of Lilium flower aroma types and identification, polymorphisms, and alternative splicing of their monoterpene synthase genes. Hortic. Res. 6, 1–15. 10.1038/s41438-019-0192-9 31645964 PMC6804824

[B13] Du Y. P. Bi Y. Yang F. P. Zhang M. F. Chen X. Q. Xue J.. (2017). Complete chloroplast genome sequences of Lilium: insights into evolutionary dynamics and phylogenetic analyses. Sci. Rep. 7, 5751. 10.1038/s41598-017-06210-2 28720853 PMC5515919

[B14] Du Y. P. He H. B. Wang Z. X. Li S. Wei C. Yuan X. N.. (2014a). Molecular phylogeny and genetic variation in the genus Lilium native to China based on the internal transcribed spacer sequences of nuclear ribosomal DNA. J. Plant Res. 127, 249–263. 10.1007/s10265-013-0600-4 24212402

[B15] Du Y. P. He H. B. Wang Z. X. Wei C. Li S. Jia G. X. (2014b). Investigation and evaluation of the genus Lilium resources native to China. Genet. Resour. Crop Evol. 61, 395–412. 10.1007/s10722-013-0045-6

[B16] Dubouzet J. G. Shinoda K. (1999). Phylogenetic analysis of the internal transcribed spacer region of Japanese Lilium species. Theoret. Appl. Genet. 98, 954–960. 10.1007/s001220051155

[B17] Gao Y. D. Harris A. J. He X. J. (2015). Morphological and ecological divergence of Lilium and Nomocharis within the Hengduan Mountains and Qinghai-Tibetan Plateau may result from habitat specialization and hybridization. BMC Evol. Biol. 15, 1–21. 10.1186/s12862-015-0405-2 26219287 PMC4518642

[B18] Gao Y. D. Harris A. J. Zhou S. D. He X. J. (2013). Evolutionary events in Lilium (including Nomocharis, Liliaceae) are temporally correlated with orogenies of the Q–T plateau and the Hengduan Mountains. Mol. Phylogenet. Evol. 68, 443–460. 10.1016/j.ympev.2013.04.026 23665039

[B19] Gao Y. D. Hohenegger M. Harris A. J. Zhou S. D. He X. J. Wan J. (2012). A new species in the genus Nomocharis Franchet (Liliaceae): evidence that brings the genus Nomocharis into Lilium. Plant Syst. Evol. 298, 69–85. 10.1007/s00606-011-0524-1

[B20] Hajibabaei M. Singer G. A.C. Hebert P. D.N. Hickey D. A. (2007). DNA barcoding: how it complements taxonomy, molecular phylogenetics and population genetics. Trends Genet. 23, 167–172. 10.1016/j.tig.2007.02.001 17316886

[B21] Haw S. G. Liang S. Y. (1986). The Lilies of China: The Genera Lilium, Cardiocrinum, Nomocharis and Notholirion. Portland, OR: Timber Press.

[B22] Hayashi K. Kawano S. (2000). Molecular systematics of Lilium and allied genera (Liliaceae): phylogenetic relationships among Lilium and related genera based on the rbcL and matK gene sequence data. Plant Species Biol. 15, 73–93. 10.1046/j.1442-1984.2000.00025.x

[B23] Heath T. A. Hedtke S. M. Hillis D. M. (2008). Taxon sampling and the accuracy of phylogenetic analyses. J. Syst. Evol. 46, 239–257.

[B24] Hoshino Y. Kanematsu N. Mii M. (2018). Evaluation of female gamete fertility through histological observation by the clearing procedure in Lilium cultivars. Breed. Sci. 68, 360–366. 10.1270/jsbbs.17153 30100803 PMC6081296

[B25] Huang J. Yang L. Q. Yu Y. Liu Y. M. Xie D. F. Li J.. (2018). Molecular phylogenetics and historical biogeography of the tribe Lilieae (Liliaceae): bi-directional dispersal between biodiversity hotspots in Eurasia. Ann. Bot. 122, 1245–1262. 10.1093/aob/mcy138 30084909 PMC6324749

[B26] Ikinci N. Oberprieler C. Güner A. (2006). On the origin of European lilies: phylogenetic analysis of Lilium section Liriotypus (Liliaceae) using sequences of the nuclear ribosomal transcribed spacers. Willdenowia 36, 647–656. 10.3372/wi.36.36201

[B27] Katoh K. Standley D. M. (2013). MAFFT multiple sequence alignment software version 7: improvements in performance and usability. Mol. Biol. Evol. 30, 772–780. 10.1093/molbev/mst010 23329690 PMC3603318

[B28] Kearse M. Moir R. Wilson A. Stones-Havas S. Cheung M. Sturrock S.. (2012). Geneious Basic: an integrated and extendable desktop software platform for the organization and analysis of sequence data. Bioinformatics 28, 1647–1649. 10.1093/bioinformatics/bts199 22543367 PMC3371832

[B29] Kim H. T. Lim K. B. Kim J. S. (2019). New insights on Lilium phylogeny based on a comparative phylogenomic study using complete plastome sequences. Plants 8, 547. 10.3390/plants8120547 31783625 PMC6963401

[B30] Kim J. H. Lee S. I. Kim B. R. Choi I. Y. Ryser P. Kim N. S. (2017). Chloroplast genomes of Lilium lancifolium, *L. amabile, L. callosum*, and *L. philadelphicum*: Molecular characterization and their use in phylogenetic analysis in the genus *Lilium* and other allied genera in the order Liliales. PLoS ONE 12, e0186788. 10.1371/journal.pone.0186788 29065181 PMC5655457

[B31] Kumar S. Stecher G. Tamura K. (2016). MEGA7: molecular evolutionary genetics analysis version 7.0 for bigger datasets. Mol. Biol. Evol., 1870–1874. 10.1093/molbev/msw054 PMC8210823 27004904

[B32] Lee C. S. Kim S. C. Yeau S. H. Lee N. S. (2011). Major lineages of the genus Lilium (Liliaceae) based on nrDNA ITS sequences, with special emphasis on the Korean species. J. Plant Biol. 54, 159–171. 10.1007/s12374-011-9152-0

[B33] Leggett R. M. Ramirez-Gonzalez R. H. Clavijo B. Waite D. Davey R. P. (2013). Sequencing quality assessment tools to enable data-driven informatics for high throughput genomics. Front. Genet. 4, 288. 10.3389/fgene.2013.00288 24381581 PMC3865868

[B34] Li J. Cai J. Qin H.-H. Price M. Zhang Z. Yu Y.. (2021). Phylogeny, age, and evolution of tribe Lilieae (Liliaceae) based on whole plastid genomes. Front. Plant Sci. 12, 699226–699226. 10.3389/fpls.2021.699226 35178055 PMC8845482

[B35] Liang S. Tamura M. (2000). “Lilium,” in Flora of China, Vol 24, ed R. P. Wu (Beijing/St. Louis: Science Press/Missouri Botanical Garden Press), 135–159.

[B36] Liang S. Y.. (1980). “Lilium L,” in Flora Reipublicae Popularis Sinicae vol. 14, Anagiospermae, Monocotyledoneae Liliaceae (I), eds F. Wang, and J. Tang (Beijing: Science Press), 116–157.

[B37] Liang S. Y.. (1995). Chorology of Liliaceae (S. Str.) and its bearing on the Chinese Flora. J. Syst. Evol. 33, 27–51.

[B38] Lighty R.. (1968). Evolutionary trends in lilies. R. Hortic. Soc. Lily Year Book 31, 40–44.

[B39] Lim K. B. Barba-Gonzalez R. Zhou S. J. Ramanna M. S. Van Tuyl J. M. (2008). Interspecific hybridization in lily (Lilium): taxonomic and commercial aspects of using species hybrids in breeding. Floric. Ornament. Plant Biotechnol. 5, 146–151. Available online at: http://s.dic.cool/S/GSoyGSQw

[B40] Lim K. B. Van Tuyl J. M. (2007). “Lily,” in Flower Breeding and Genetics, ed N. O. Anderson (Dordrecht: Springer), 517–537.

[B41] Liu C. Q. Gao Y. D. Niu Y. Xiong Y. Z. Sun H. (2019). Floral adaptations of two lilies: implications for the evolution and pollination ecology of huge trumpet-shaped flowers. Am. J. Bot. 106, 622–632. 10.1002/ajb2.1275 31022316

[B42] Madhavan S. Balasubramanian V. Selvarajan R. (2021). “Viruses infecting bulbous ornamental plants and their diagnosis and management,” in Virus Diseases of Ornamental Plants, eds S. K. Raj, R. K. Gaur, and Z. Yin (Singapore: Springer), 277–299.

[B43] McRae E. A.. (1998). Lilies: A Guide for Growers And Collectors. Portland, OR: Timber Press.

[B44] Miller W. B.. (2017). Flower bulbs worldwide: perspectives on the production chain and research. Acta Hortic. 1171, 1–8. 10.17660/ActaHortic.2017.1171.1

[B45] Mitchell R.. (1998). Species DNA research report. NALS Q. Bull. 52, 8–9.

[B46] Muratović E. Hidalgo O. Garnatje T. Siljak-Yakovlev S. (2010). Molecular phylogeny and genome size in European lilies (Genus Lilium, Liliaceae). Adv. Sci. Lett. 3, 180–189. 10.1166/asl.2010.1116

[B47] Nikiforova S. V. Cavalieri D. Velasco R. Goremykin V. (2013). Phylogenetic analysis of 47 chloroplast genomes clarifies the contribution of wild species to the domesticated apple maternal line. Mol. Biol. Evol. 30, 1751–1760. 10.1093/molbev/mst092 23676769

[B48] Nishikawa T. Okazaki K. Arakawa K. Nagamine T. (2001). Phylogenetic analysis of section Sinomartagon in genus *Lilium* using sequences of the internal transcribed spacer region in nuclear ribosomal DNA. Breed. Sci. 51, 39–46. 10.1270/jsbbs.51.39

[B49] Nishikawa T. Okazaki K. Uchino T. Arakawa K. Nagamine T. (1999). A molecular phylogeny of Lilium in the internal transcribed spacer region of nuclear ribosomal DNA. J. Mol. Evol. 49, 238–249. 10.1007/PL00006546 10441675

[B50] Patterson T. B. Givnish T. J. (2002). Phylogeny, concerted convergence, and phylogenetic niche conservatism in the core Liliales: insights from rbcL and ndhF sequence data. Evolution 56, 233–252. 10.1111/j.0014-3820.2002.tb01334.x 11926492

[B51] Pelkonen V. P. Pirttilä A. M. (2012). Taxonomy and phylogeny of the genus Lilium. Floricult. Ornam. Biotechnol. 6, 1–8. Available online at: http://s.dic.cool/S/psIyvGaH

[B52] Pfenninger M. Nowak C. Kley C. Steinke D. Streit B. (2007). Utility of DNA taxonomy and barcoding for the inference of larval community structure in morphologically cryptic Chironomus (Diptera) species. Mol. Ecol. 16, 1957–1968. 10.1111/j.1365-294X.2006.03136.x 17444904

[B53] Qu X. J. Moore M. J. Li D. Z. Yi T. S. (2019). PGA: a software package for rapid, accurate, and flexible batch annotation of plastomes. Plant Methods 15, 1–12. 10.1186/s13007-019-0435-7 31139240 PMC6528300

[B54] Rabah S. O. Shrestha B. Hajrah N. H. Sabir M. J. Alharby H. F. Sabir M. J.. (2019). Passiflora plastome sequencing reveals widespread genomic rearrangements. J. Syst. Evol. 57, 1–14. 10.1111/jse.12425

[B55] Reichenbach H. G.L.. (1830). Flora germanica Excursoria ex Affinitate Regni Vegetabilis Naturali Disposita, Sive Principia Synopseos Plantarum in Germania terrisque in Europa Media Adjacentibus Sponte Nascentium Cultarumque Frequentius. Lipsiae: Carolum Cnobloch.

[B56] Ronquist F. Teslenko M. Van Der Mark P. Ayres D. L. Darling A. Höhna S.. (2012). MrBayes 3.2: efficient Bayesian phylogenetic inference and model choice across a large model space. Syst. Biol. 61, 539–542. 10.1093/sysbio/sys029 22357727 PMC3329765

[B57] Stamatakis A.. (2014). RAxML version 8: a tool for phylogenetic analysis and post-analysis of large phylogenies. Bioinformatics 30, 1312–1313. 10.1093/bioinformatics/btu033 24451623 PMC3998144

[B58] Tang Y. C. Liu Y. J. He G. R. Cao Y. W. Bi M. M. Song M.. (2021). Comprehensive analysis of secondary metabolites in the extracts from different lily bulbs and their antioxidant ability. Antioxidants 10, 1634. 10.3390/antiox10101634 34679768 PMC8533310

[B59] Valcárcel V. Wen J. (2019). Chloroplast phylogenomic data support Eocene amphi-Pacific early radiation for the Asian Palmate core Araliaceae. J. Syst. Evol. 57, 547–560. 10.1111/jse.12522

[B60] Van Creij M. G. M. Van Raamsdonk L. W. D. Van Tuyl J. M. (1990). Wide Interspecific Hybridization of Lilium: Preliminary Results of the Application of Pollination and Embryo-Rescue Methods. New York, NY: Lily Yearbook of the North American Lily Society, Inc., 28–37.

[B61] Van Tuyl J. M. Arens P. Ramanna M. S. Shahin A. Khan N. Xie S.. (2011). “Lilium,” in Wild Crop Relatives: Genomic and Breeding Resources, eds A. Grassotti and G. Burchi (Berlin, Heidelberg: Springer), 161–183.

[B62] Van Tuyl J. M. Arens P. Shahin A. Marasek-Ciołakowska A. Barba-Gonzalez R. Kim H. T.. (2018). “Lilium,” in Ornamental Crops. Handbook of Plant Breeding, ed J. Van Huylenbroeck (Cham: Springer), 481–512.

[B63] Van Tuyl J. M. Van Diën M. P. Van Creij M. Van Kleinwee T. Franken J. Bino R. (1991). Application of in vitro pollination, ovary culture, ovule culture and embryo rescue for overcoming incongruity barriers in interspecific Lilium crosses. Plant Sci. 74, 115–126. 10.1016/0168-9452(91)90262-7

[B64] Viljoen E. Odeny D. A. Coetzee M. P. Berger D. K. Rees D. J. (2018). Application of chloroplast phylogenomics to resolve species relationships within the plant genus Amaranthus. J. Mol. Evol. 86, 216–239. 10.1007/s00239-018-9837-9 29556741

[B65] Wang H. X. Moore M. J. Barrett R. L. Landrein S. Sakaguchi S. Maki M.. (2020). Plastome phylogenomic insights into the Sino-Japanese biogeography of Diabelia (Caprifoliaceae). J. Syst. Evol. 58, 972–987. 10.1111/jse.12560

[B66] Wen J. Herron S. A. Yang X. Liu B. B. Zuo Y. J. Harris A.. (2020). Nuclear and chloroplast sequences resolve the enigmatic origin of the concord grape. Front. Plant Sci. 11, 263. 10.3389/fpls.2020.00263 32256506 PMC7092692

[B67] Wilson E. H.. (1925). Lilies of Eastern Asia. London: Dulau & Company, Ltd.

[B68] Zhang N. Erickson D. L. Ramachandran P. Ottesen A. R. Timme R. E. Funk V. A.. (2017). An analysis of Echinacea chloroplast genomes: implications for future botanical identification. Sci. Rep. 7, 1–9. 10.1038/s41598-017-00321-6 28303008 PMC5428300

[B69] Zhang X. Zhang H. J. Landis J. B. Deng T. Meng A. P. Sun H.. (2019). Plastome phylogenomic analysis of Torreya (Taxaceae). J. Syst. Evol. 57, 607–615. 10.1111/jse.1248225084786

[B70] Zhao Y. Yin J. Guo H. Zhang Y. Xiao W. Sun C.. (2015). The complete chloroplast genome provides insight into the evolution and polymorphism of Panax ginseng. Front. Plant Sci. 5, 696. 10.3389/fpls.2014.00696 25642231 PMC4294130

[B71] Zwickl D. J. Hillis D. M. (2002). Increased taxon sampling greatly reduces phylogenetic error. Syst. Biol. 51, 588–598. 10.1080/10635150290102339 12228001

